# Multi-modal 3D imaging of radionuclides using multiple hybrid Compton cameras

**DOI:** 10.1038/s41598-022-06401-6

**Published:** 2022-02-15

**Authors:** Akihisa Omata, Miho Masubuchi, Nanase Koshikawa, Jun Kataoka, Hiroki Kato, Atsushi Toyoshima, Takahiro Teramoto, Kazuhiro Ooe, Yuwei Liu, Keiko Matsunaga, Takashi Kamiya, Tadashi Watabe, Eku Shimosegawa, Jun Hatazawa

**Affiliations:** 1 Graduate School of Advanced Science and Engineering, Waseda University, Tokyo, Japan; 2 Graduate School of Medicine, Osaka University, Osaka, Japan; 3 Institute for Radiation Sciences, Osaka University, Osaka, Japan

**Keywords:** Cancer imaging, Experimental nuclear physics, Imaging techniques, Molecular medicine

## Abstract

For radiological diagnosis and radionuclide therapy, X-ray and gamma-ray imaging technologies are essential. Single-photon emission tomography (SPECT) and positron emission tomography (PET) play essential roles in radiological diagnosis, such as the early detection of tumors. Radionuclide therapy is also rapidly developing with the use of these modalities. Nevertheless, a limited number of radioactive tracers are imaged owing to the limitations of the imaging devices. In a previous study, we developed a hybrid Compton camera that conducts simultaneous Compton and pinhole imaging within a single system. In this study, we developed a system that simultaneously realizes three modalities: Compton, pinhole, and PET imaging in 3D space using multiple hybrid Compton cameras. We achieved the simultaneous imaging of Cs-137 (Compton mode targeting 662 keV), Na-22 (PET mode targeting 511 keV), and Am-241 (pinhole mode targeting 60 keV) within the same field of view. In addition, the imaging of Ga-67 and In-111, which are used in various diagnostic scenarios, was conducted. We also verified that the 3D distribution of the At-211 tracer inside a mouse could be imaged using the pinhole mode.

## Introduction

In the nuclear medicine field, it is essential to visualize the distribution of radioisotopes in a patient’s body. Particularly, a radiological diagnosis that enables non-invasive visualization of the affected region from outside the body is vital for detecting early stages of diseases and other medical conditions^1^. Moreover, nuclear medicines involving radioactive sources are used to treat diseases, such as cancer, demanding drug visualization^2,3^. The general approach is to visualize the nuclear gamma rays emitted from radioactive tracers. Single-photon emission computed tomography (SPECT) and positron emission tomography (PET) are widely used for various diagnoses^4,5^. However, the use of SPECT and PET is limited to a specific energy range of either X-rays or gamma rays, whereas the photons from radionuclides have a wide energy range from a few keV to several MeV. Moreover, the application of PET is limited to a positron emitter. This leads to a limited number of radioactive tracers that can be imaged using current SPECT and PET scanners. In this context, a Compton camera^6,7^ that can perform imaging in a wide energy band is crucial. Compton cameras are capable of efficiently imaging high-energy photons^8,9^, and therefore have been investigated for medical applications, such as visualization of 4.4 MeV prompt gamma rays toward monitoring for proton therapy^10–14^. Furthermore, for clinical applications, both scintillator-based and semiconductor-based Compton cameras have been developed^15–21^. Several studies have demonstrated the feasibility of in vivo mouse imaging using a Compton camera^22–26^. Semiconductor-based Compton cameras have excellent energy resolutions. In contrast, scintillator detectors easily assemble such a unique structure and have a high Z, which directly corresponds to a high efficiency and cost-effectiveness. However, scintillator-based Compton cameras are generally unsuitable for low-energy photon imaging because the dominant interaction for lower-energy photons is photo-absorption rather than Compton scattering. As described above, presently three different techniques are used in medical imaging, namely, SPECT, PET, and Compton cameras, to enable imaging of a wide range of photon energies emitted by radionuclides. Nevertheless, these scanners and cameras are expensive and additional measurement time is required for use in clinical situations. Hence, a technology that produces broadband energy in the range of several tens of keV to MeV is required to accelerate improvements in diagnosis and therapy.

Several approaches have been proposed to achieve broadband imaging. One approach is to extend the energy range of the existing principle by examining the material and/or configuration of the detector. For instance, to adapt SPECT to high-energy photons, the configuration of the collimator or a method to analytically compensate for scattering components have been studied^27–29^. A Compton camera using Si/CdTe semiconductors has been reported to improve the sensitivity to lower-energy photons^20,23^, which realized the simultaneous capture of F-18 FDG and Tc-99m DMSA^30,31^. Another approach is to conduct imaging based on two different principles in a single detection system. Yoshida et al. (2020) described whole gamma imaging (WGI) as a combination of a PET scanner and Compton camera^32^. In our previous study, we proposed a hybrid Compton camera (HCC) that realizes simultaneous wide-band imaging that combines the advantages of a Compton camera and pinhole camera in a single detector system^33^. However, a simple imaging system that encompasses photons emitted by radionuclides from a few keV to several MeV is still challenging.

In this study, we developed a system consisting of four HCCs to extend pinhole/Compton imaging to 3D space. Furthermore, PET imaging was enabled by extracting simultaneous detection events between multiple HCCs. In total, we propose 3D imaging using three imaging modalities: pinhole imaging, Compton imaging, and PET imaging. In addition, we experimentally confirmed the simultaneous 3D imaging of Am-241 (60 keV; pinhole mode), Cs-137 (662 keV; Compton mode), and Na-22 (annihilation photons; PET mode). Each reconstruction mode was selected after measurement. Furthermore, experiments were performed for applications in nuclear medicine. We succeeded in locating the Ga-67 and In-111 sources in 3D space. Subsequently, imaging of a mouse with At-211 was successfully performed. Also, for clinical applications, the effect of absorption and scattering of photons in the patient’s body is an issue to be considered. To mimic the situation, we evaluated the effects of scattering and absorption using a water phantom placed between the camera and the Ba-133 source and estimated the thickness of water by analyzing the spectral features.

**Figure 1:**
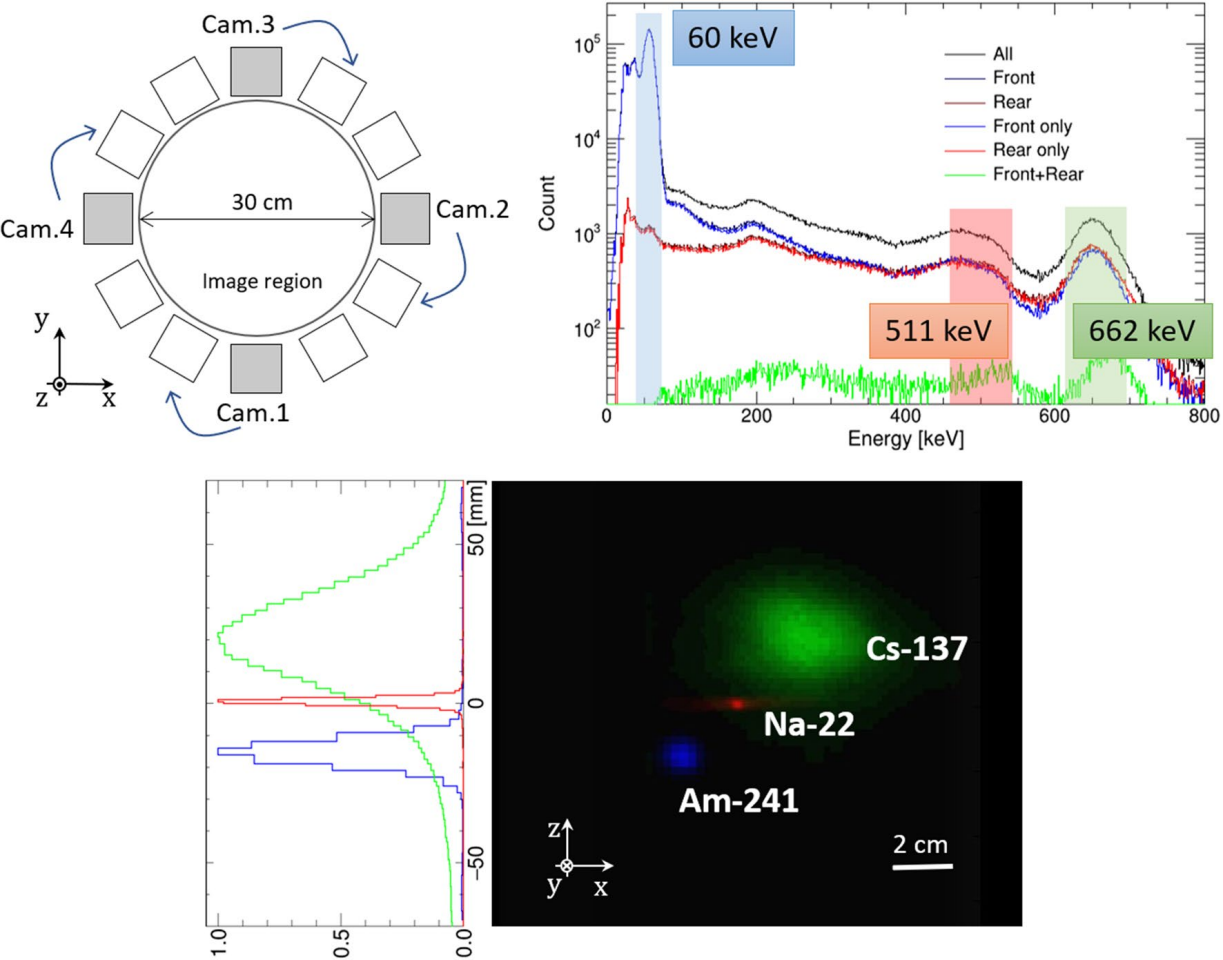
(*Upper left*) Configuration of the multi-angle measurement. (*upper right*) Energy spectrum obtained via a HCC by using Cs-137, Na-22, and Am-241 sources simultaneously. (*lower*) Slice of the 3D reconstructed image (*right*) and the projections of each source (*left*); green, red, and blue conversions correspond to Cs-137 (662 keV; Compton mode), Na-22 (PET mode), and Am-241 (60 keV; pinhole mode) sources, respectively.

## Results and discussion

### Demonstration of the multi-modal imaging

The performance of HCCs as a multi-modal 3D imager was first demonstrated by simultaneous imaging of Cs-137 (904 kBq), Na-22 (45 kBq), and Am-241 (3.93 MBq) sources. The Cs-137 and Na-22 sources were point-like and the Am-241 source had a diameter of less than 1 cm. We adopted multi-angle data acquisition^24^ using four HCCs. The opposite HCCs were placed 30 cm away from each other, and the three radiation sources were set at the center of the cameras, as shown in Fig. 1 (*upper left*). The Na-22 source was placed at the center of HCCs ($x=0$, $y=0$, $z=0$), Cs-137 at $x=2$ cm, $y=0$ cm, $z=2$ cm, and Am-241 at $x=-2$ cm, $y=0$ cm, $z=-2$ cm. The measurements were taken three times after rotating the sources 30 $^\circ$ each time, which corresponds to a total of 12 angles. Figure 1 (*upper right*) shows the energy spectrum obtained from an angle. The Cs-137 (662 keV), Na-22 (positron), and Am-241 (60 keV) sources were reconstructed in the Compton, PET, and pinhole modes, respectively. The number of selected events for Compton, PET, and pinhole modes were 15,827, 8,449, and 479,096, respectively. Multi-color 3D images of the three sources were acquired by projecting them to the same coordinates. As shown in Fig. 1 (*lower*), each convergence group indicates the correct positions, underlining the potential of multi-modal 3D imaging using HCCs. We note that, in the case of multiple sources being imaged simultaneously, nuclides other than the target nuclide for each modality may contaminate as background signals. These backgrounds are dependent on the type and intensity of nuclides, as shown by Kishimoto et al.^24^. To reduce this background, we are developing a new detector system, that is, HCC shielded with BGO scintillators. In this concept, BGO detectors act as an “active shield” of HCC such that incident gamma rays, depositing only a part of their energy but penetrating through the absorber, are effectively removed; thus, the contamination can be substantially reduced. The full details of the new BGO-shielded detector are discussed elsewhere.

Additionally, Table 1 shows the absolute efficiencies of each mode obtained via a Monte Carlo simulation^34^ as compared with the experimental values. The simulation and experimental configuration was the same as that shown in Fig. 1 (*upper left*); a total of 12 HCCs were positioned in a ring with a monochromatic source (60 keV, 662 keV point source, and 511-keV annihilation photons, independently) at the center of the HCCs. The efficiency values were calculated and measured independently for each source. The absolute efficiency values indicate the fraction of events detected as each mode with respect to the total radiation emitted from the source. There are several possible reasons for the difference between the simulated efficiency and the measured efficiency. One is that the time resolution, which corresponds to the coincidence window, is not considered in the simulation; this is one reason for the overestimation of the PET modality. Furthermore, although the nuclide actually emits several photon energies during the decay process, only the target energy photons are irradiated in the simulation, causing an underestimation of the Compton modality. The current protocol has deviations in resolution and sensitivity depending on the mode; however, resolution and sensitivity can be optimized according to the application and purpose.

**Table 1:** Summary of simulated and experimented absolute efficiencies of each mode.

Targeting source	Reconstruction mode	Simulated efficiency	Measured efficiency
Cs-137 (662 keV)	Compton	$(1.52 \pm 0.08) \times 10^{-5}$	$(1.87 \pm 0.07) \times 10^{-5}$
Am-241 (60 keV)	Pinhole	$(5.21 \pm 0.16) \times 10^{-5}$	$(4.98 \pm 0.06) \times 10^{-5}$
Na-22 (511 keV)	PET	$(4.47 \pm 0.01) \times 10^{-4}$	$(3.34 \pm 0.05) \times 10^{-4}$

### Ga-67/In-111 imaging of a small bottle

**Figure 2:**
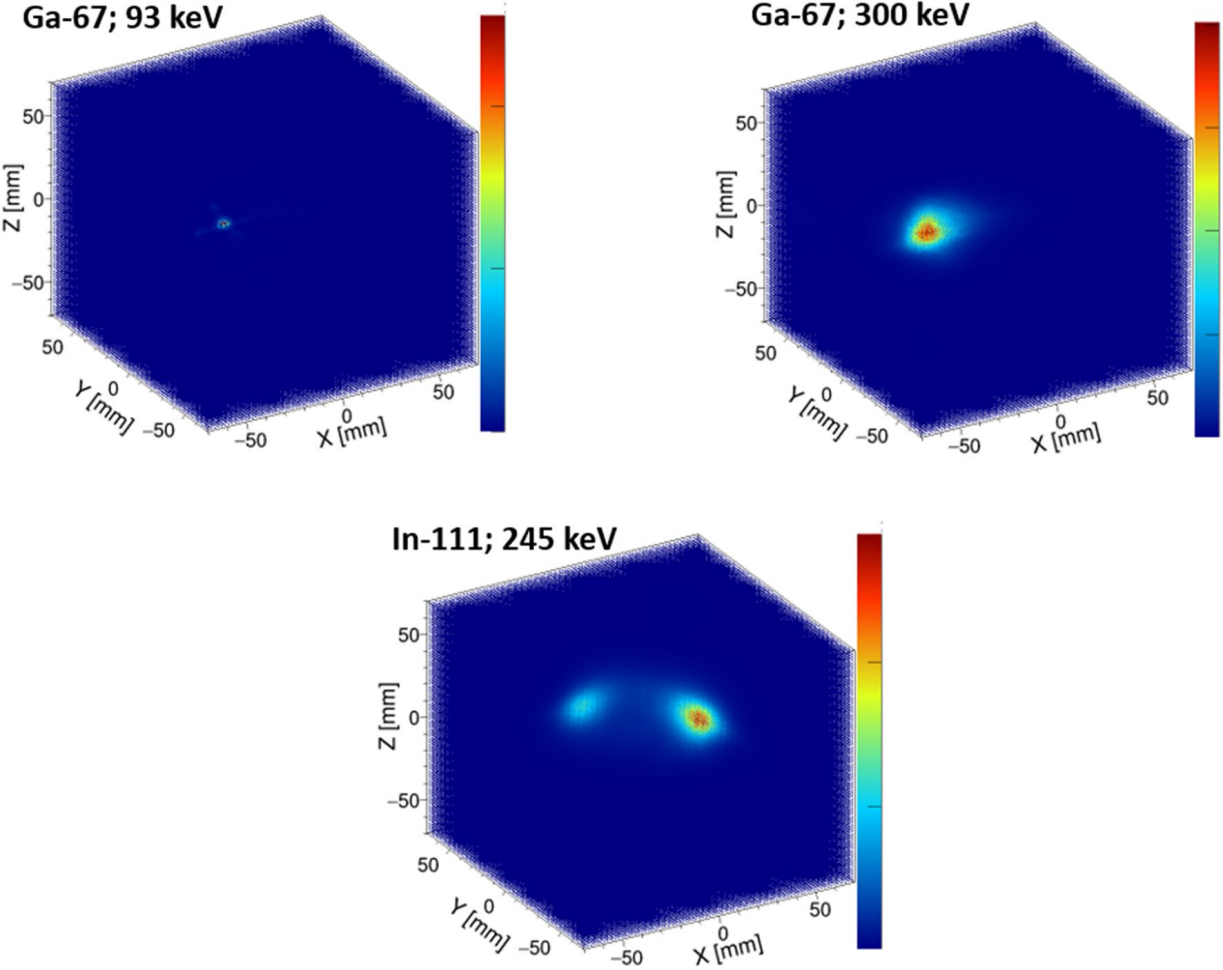
Pinhole (*upper left*; 93 keV) and Compton (*upper right*; 300 keV) MLEM images of a bottle with Ga-67 in 3D space. (*lower*) Compton MLEM images of two bottles with In-111 (245 keV) in 3D space.

Furthermore, we imaged Ga-67 and In-111 sources, which are used in various diagnostic scenarios^35–37^. Initially, a bottle with a Ga-67 source (0.25 MBq) was imaged. The source was approximately 200 μL and was enclosed in a microtube. The Ga-67 source was point-like (diameter < 1 cm) and surrounded by the four HCCs placed 30 cm away from each other. The actual location of the Ga-67 source was $x=-30$ mm, $y=30$ mm, $z=-7$ mm. The measurement time was 30 min, resulting in 113k pinhole events targeted at 93 keV and 4.1 k Compton events targeted at 300 keV. Figure 2 (*upper*) shows the reconstructed pinhole (*left*) and Compton (*right*) images in 3D space.

Next, two bottles of In-111 source (0.27 MBq and 0.36 MBq) were imaged. The sources were approximately 200 $\mathrm{\mu }$L and were enclosed in microtubes, respectively. The HCCs were placed around the source, 30 cm apart from each camera as shown in Fig. 1 (*upper left*) and then rotated by 45 $^\circ$; the total measurement angles were eight. The In-111 sources were point-like (diameter $< 1$ cm) and the actual locations were $x=-30$ mm, $y=30$ mm, $z=35$ mm, and $x=30$ mm, $y=-30$ mm, $z=35$ mm. The 30 min measurement for each setting resulted in 79k Compton events targeted at 245 keV. The MLEM Compton reconstructed image is shown in Fig. 2 (*lower*). The ratio of the integrated pixel values in the region of the two In-111 sources was 0.80 ± 0.13, which reproduced an actual intensity ratio of 0.75.

### At-211 imaging of a mouse

**Figure 3:**
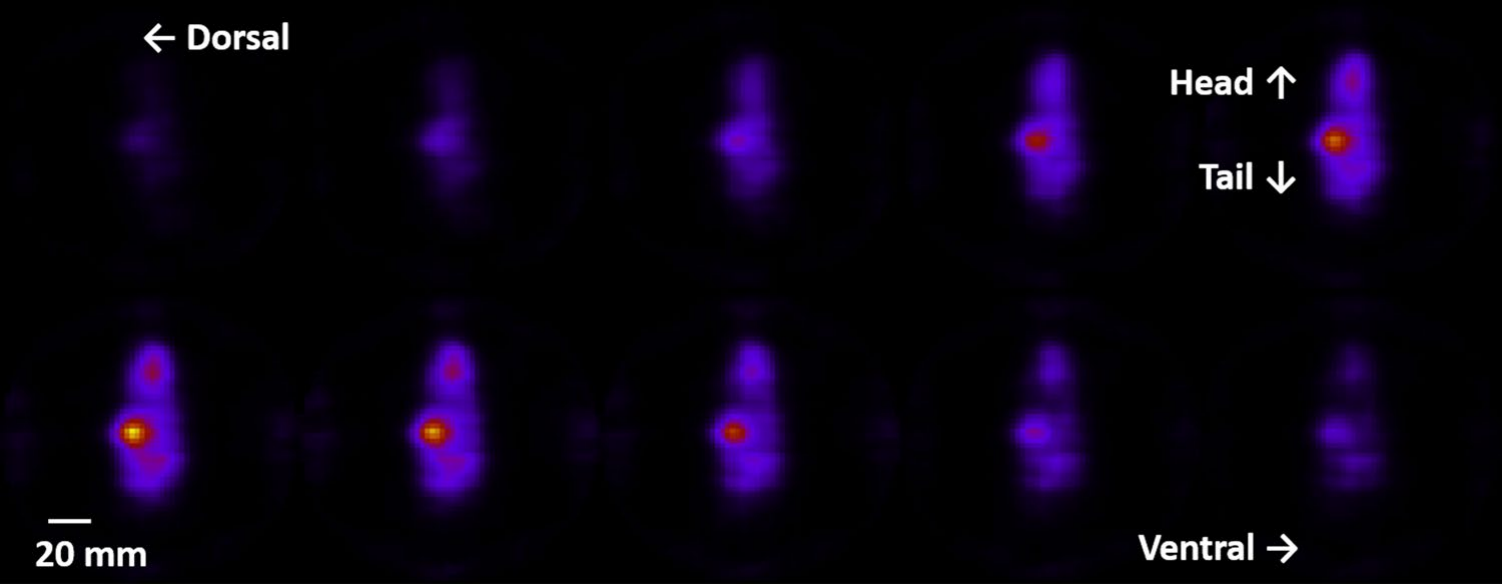
2D slices of the 3D reconstructed image (79 keV; pinhole mode) of the mouse with At-211. Each figure shows a 2.3-mm-pitch slice from the dorsal to the ventral side.

For applications in future nuclear medicine, we investigated the capability of our multiple HCC system using a mouse with an At-211 tracer. At-211 has been designated for use as a source of alpha particles for radionuclide therapy^38,39^. It emits intense characteristic X-rays (mainly 79 keV) and weak nuclear gamma rays (570, 687, and 898 keV) during α decay. The mouse was injected with At-211 (0.96 MBq) and euthanized by an overdose of isoflurane 3 h after injection. The HCCs were placed around the mouse in the standing position, as shown in Fig. 1 (*upper left*), and data were collected from 12 angles by rotating the mouse. The measurement time was 40 min for each angle, resulting in 218,745 pinhole events. Figure 3 shows the 2D slices of the pinhole 3D reconstruction image obtained from 12 angles. The pinhole image shows that the 3D-space distribution of At-211 converges on the thyroid and stomach. The number of Compton events (570 keV) was 438, which was consistent with the expected value; however, the number of events is too small to reproduce the source distribution in three dimensions.

### Effect of the body components

**Figure 4:**
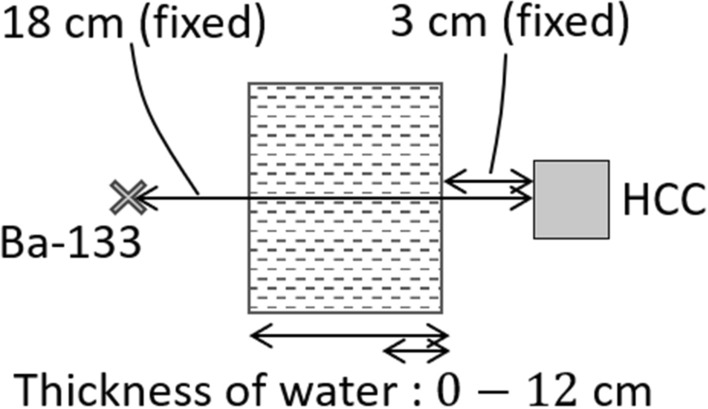
Measurement configurations with various thicknesses of water.

In a clinical situation, drugs are distributed inside a patient’s body and are affected by scattering due to the tissues that compose the body. Hereafter, the composition of the body is replaced with water when discussing the effect of scattering. Specifically, as a representative clinical situation, the data were acquired in a situation where different thicknesses were present between the point-like Ba-133 source and the camera. As shown in Fig. 4, the distance from the Ba-133 source to the HCC was constant (18 cm), and the thickness of water was varied: 0, 2, 4, 6, 8, 10, and 12 cm. The position of the water surface closest to the HCC was fixed. The measurement time was 30 min for each. We describe the effect of scattering using reconstructed images and spectra.

**Figure 5:**
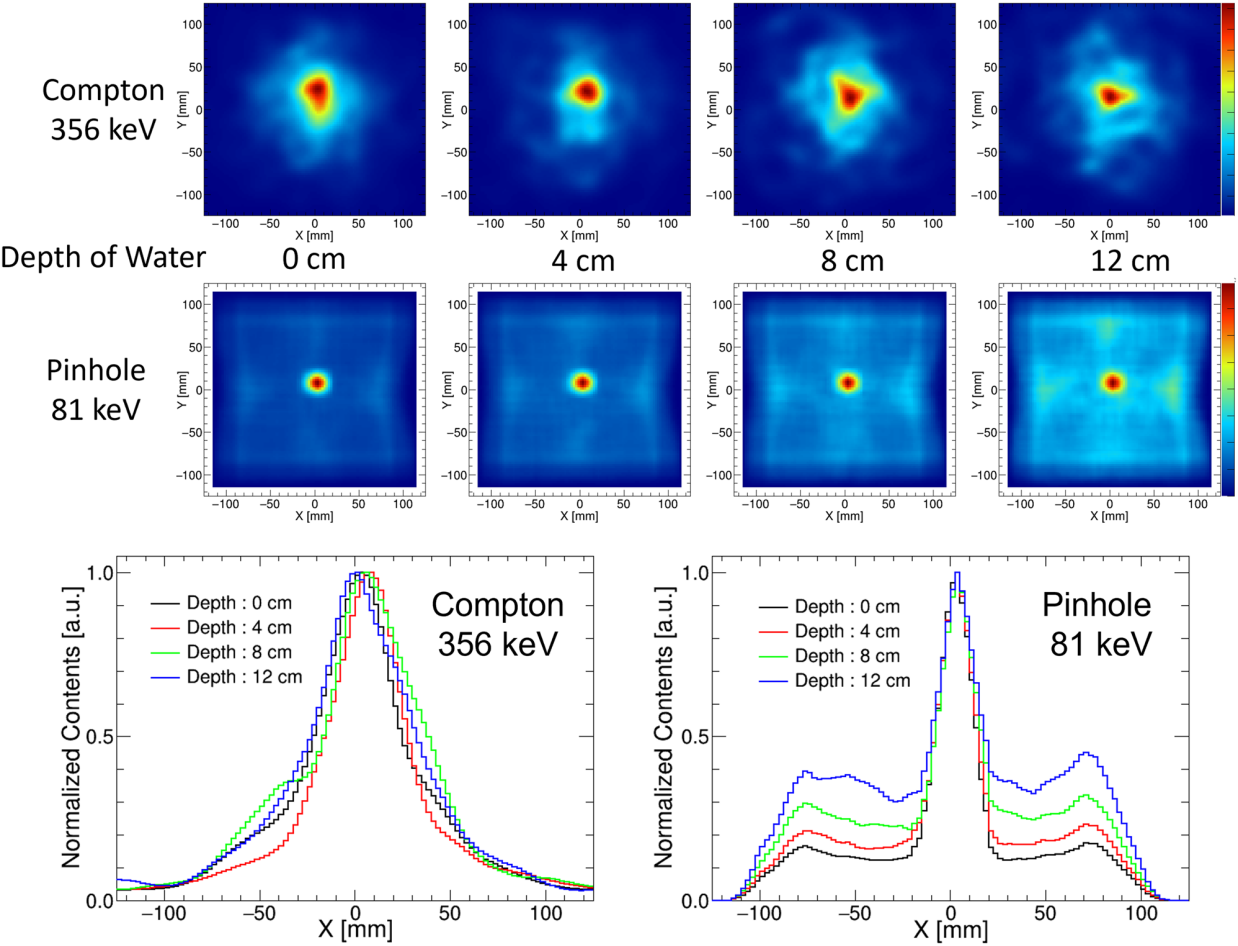
2D reconstructed images for different thicknesses of water (*upper two rows*) and the projection of those images (*lower*).

Figure 5 shows the reconstructed images at each thickness and its projection image. Compton reconstructed images (targeting 356 keV) were comparable at all thicknesses. Meanwhile, in the pinhole reconstruction images (targeting 81 keV), the offset component increased as the thickness of water increased. This is because the reaction cross section for Compton scattering of photons in water is higher at a lower energy level of 81 keV.

**Figure 6:**
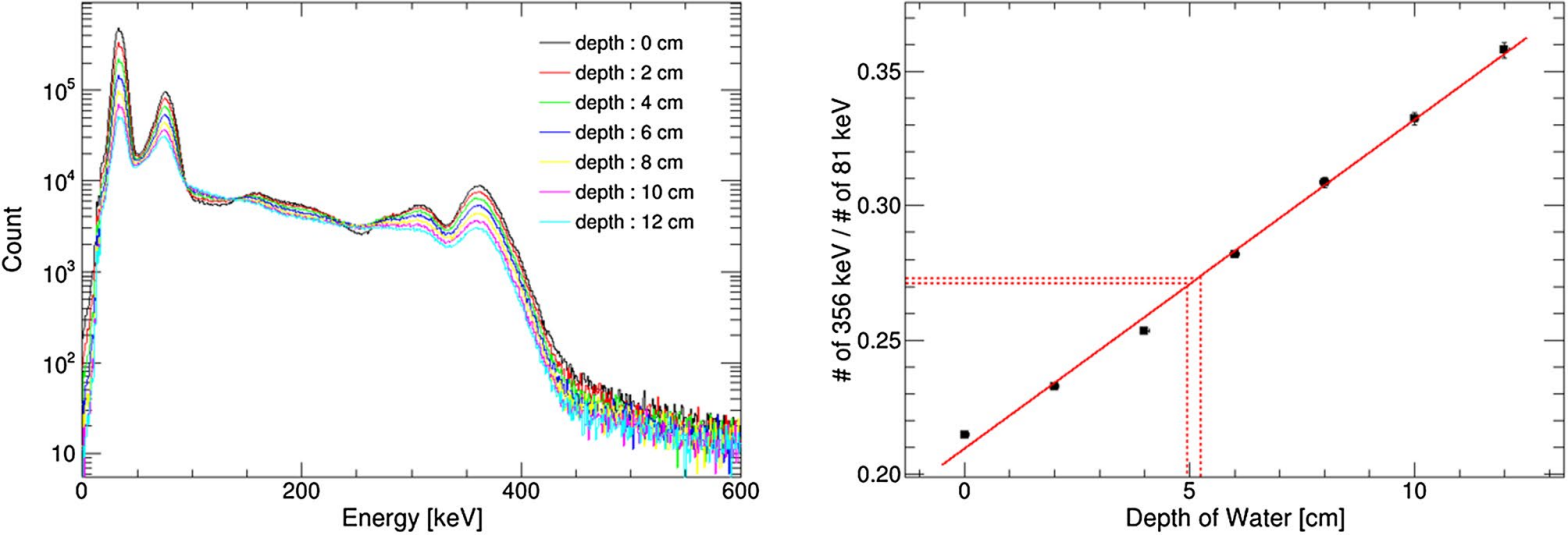
(*Left*) Spectrum obtained with different thicknesses of water. (*right*) Correlation between the water thickness and ratio of photopeaks.

Next, we attempted to estimate the thickness of water by analyzing the spectral features. Iwamoto et al. reported a method for estimating the thickness of materials based on the modulation of the photopeak to scattering component ratio^40^. We used the variation in the ratio of photopeaks of different energies. This is because the attenuation coefficient of a material is correlated with photon energy. Therefore, we can estimate the thickness of the material, which corresponds to the thickness information, using the modulation indicated by the ratio of photopeaks. The spectra obtained in each measurement indicate the variation in the intensity of the photopeak with respect to the thickness of water, as shown in Fig. 6 (*left*). Figure 6 (*right*) shows the transition of the ratio of photopeaks at 81 keV and 356 keV, which indicates a tight correlation between water thickness and the photopeak ratio. Subsequently, we attempted to estimate the water thickness between the source and camera. The measurement configurations are shown in Fig. 4. The thickness of water was estimated using the table of correlations between the thickness and photopeak ratios. The estimated thickness of water matched the actual thickness, as shown in Fig. 6 (*right*; red dashed line) and Table 2.

**Table 2:** Estimation of the thickness of water.

Measured ratio	$0.272 \pm 0.001$
Estimated thickness (cm)	$5.08 \pm 0.15$
Actual thickness (cm)	5

## Methods

### Detector configuration

**Figure 7:**
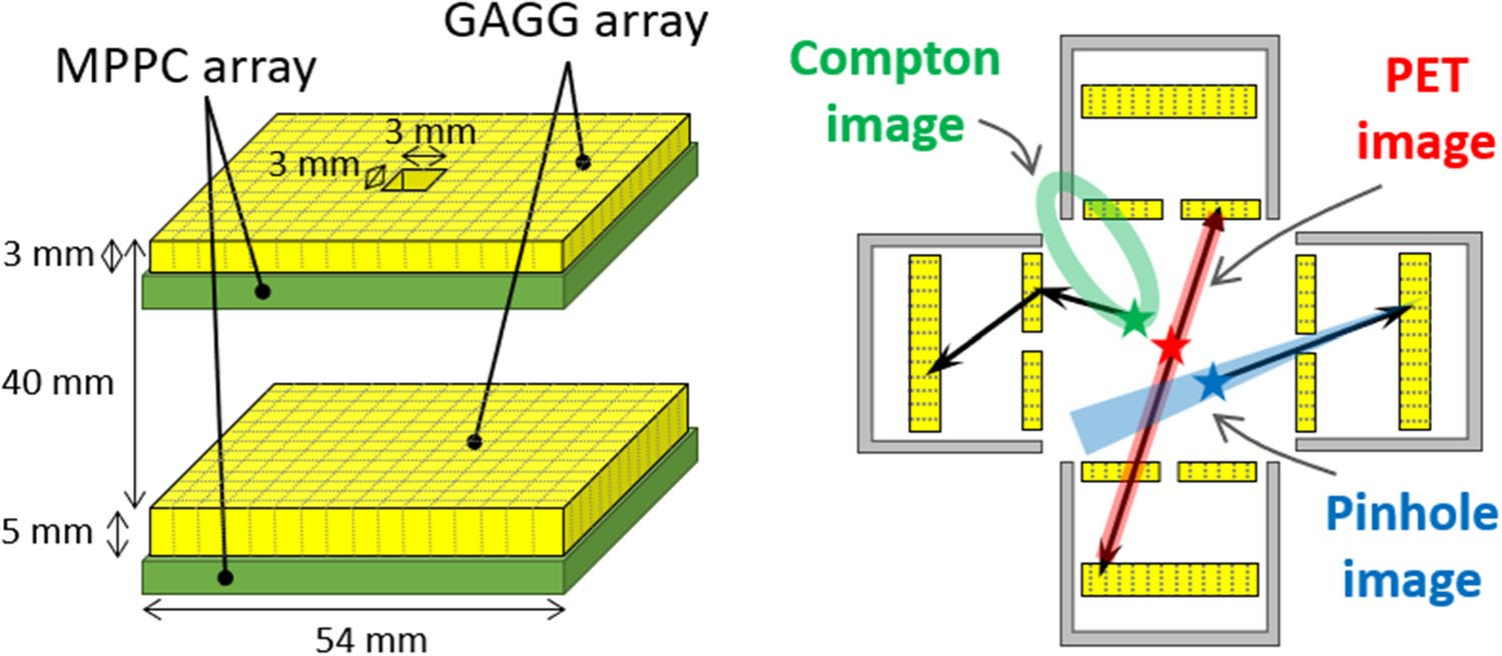
(*Left*) Configuration of the hybrid Compton camera (HCC). (*right*) Schematic of reconstruction in multi-modality: Compton, PET, and pinhole imaging.

We developed a novel system consisting of four HCCs. Each camera in the system is similar to that reported by Omata et al.^33^ except for the size of the pinhole. As shown in Fig. 7 (*left*), the cameras consist of a pair of position-sensitive detectors capable of acquiring the reaction position and energy deposit with time information for each event. Both detectors are composed of Ce-doped $\mathrm{Gd}_3\mathrm{Al}_2\mathrm{Ga}_3\mathrm{O}_{12}$ scintillator arrays^41,42^ coupled with multi-pixel photon counter (MPPC) arrays. The front scintillator is a $45\times 45$ array, with each pixel being $1\times 1\times 3\ \mathrm{mm^3}$ in size. The rear scintillator is a $45\times 45$ array, with each pixel being $1\times 1\times 5\ \mathrm{mm^3}$ in size. The distance between the front detector and rear detector is 40 mm. The front detector has a pinhole of $3\times 3$ $\mathrm{mm}^2$ in its center to act as an active pinhole shield. The energy resolutions (full width at half maximum) of each pixel for a given array are 7.3 ± 0.8 % at 662 keV, 7.8 ± 0.8 % at 511 keV, and 22.8 ± 1.2 % at 60 keV. The cameras are covered with a 3 mm-thick heavy metal (mainly tungsten; density 18.0 g/cm^3^) case, except for the front surface. All events are marked with timestamp information common to all four camera systems.

Each HCC enables both Compton and pinhole imaging with a single detector system using the front detector as a scatterer for high-energy photons (>200 keV) and active pinhole for low-energy photons (< 200 keV). In other words, for high-energy photons, the events that are scattered in the front detector and subsequently absorbed in the rear detector can be used for Compton imaging; for low-energy photons, the events arriving at the rear detector can be used for pinhole imaging because the photons are shielded, except for the hole in the front detector. Furthermore, by using multiple HCCs for stereo imaging, we achieved both Compton and pinhole imaging in 3D space. Additionally, PET imaging was performed by measuring a pair of 511 keV photons simultaneously acquired at different HCCs. Figure 7 (*right*) shows a schematic of each mode.

**Table 3:** Factors of event selection for each reconstruction mode.

Candidate	Coincidence	Energy
Compton	Front&& rear	(Front + rear) and front
Pinhole	(Not front)&& rear	Rear
PET	Cam.X&& Cam.Y	Front + rear

### Multi-modal reconstruction

The elements for selecting the events to be reconstructed for each mode are summarized in Table 3. In each reconstruction mode, the candidates for reconstruction events were selected based on the detector hit patterns. Then, an energy cut was used to restrict the energy range according to the target energy. The procedure for each reconstruction mode is carried out as follows.

For Compton reconstruction, the events that are detected simultaneously by the front and rear detectors of the same HCC are the candidates for reconstruction events. Compton events are restricted from the total energy deposit and energy deposit of the front detector. The front detector energy restriction aims to eliminate back-scattering events. During Compton reconstruction, we applied the following algorithm, which is based on list-mode maximum-likelihood expectation-maximization (MLEM)^24,43,44^:

1 $$\begin{aligned} \lambda _j^n = \lambda _j^{n-1} \sum _{k} \frac{1}{s_j^l} \frac{t_{kj} v_k}{\sum _{j^\prime } t_{k{j^\prime }} \lambda _{j^\prime }^{n-1}} \end{aligned}$$

where $\lambda _j^n$ is the reconstructed image value after the $n$th iteration, $s_j^l$ is the probability that a photon emitted from image voxel *j* is detected at a certain data acquisition angle *l*, $v_k$ is the probability that an event *k* comes from the image space, and $t_{kj}$ is the system matrix where a photon emitted from image voxel *j* will be measured as an event *k*. We applied the following equation as the system matrix $t_{kj}$, which simplified the equation described in Kishimoto et al.^24^:

2 $$\begin{aligned} t_{kj}=2\pi \Bigg (1-\frac{d}{\sqrt{d^2+a^2}}\Bigg )\times \exp \Bigg \{-\frac{1}{2}\Bigg (\frac{|\Theta _j|-|\theta _k|}{\sigma }\Bigg )^2\Bigg \}\times \frac{1}{\sin {\theta _k}} \end{aligned}$$

where *a* is the half size of the imaging voxel, *d* is the distance between the pixel reacting to Compton scattering and the interested imaging voxel *j*; $\Theta _j$ is the angle between the scattering axis and the direction of the imaging voxel *j*; $\theta _k$ is the scattering angle of the *k*th event calculated using the energy information. Here, the Gaussian width $\sigma$ in the second term corresponds to the uncertainty of the calculated scattering angle and it is better to apply a small value to avoid a double count of the uncertainty^45^. We set $\sigma$ to 2.5° for Compton reconstruction. The sensitivity matrix $S_j^l$ is calculated using a Monte Carlo simulation to irradiate the camera positioned at an angle *l* with a uniform source of radiation from the region of interest.

For pinhole reconstruction, the events that are not detected in the front detector but detected in the rear detector are selected as candidates, which ideally correspond to the events that have passed through the hole in the front detector. Then, the pinhole events are selected according to the energy cuts within the target energy range. We reconstructed the pinhole image using the following hist-mode MLEM^46,47^:

3 $$\begin{aligned} \lambda _j^n = \frac{\lambda _j^{n-1}}{\sum _i c_{ij}} \sum _{i} \frac{c_{ij} y_{i}}{\sum _{j^\prime } c_{i{j^\prime }} \lambda _{j^\prime }^{n-1}} \end{aligned}$$

where $\lambda _j^n$ is the reconstructed image value after the *n*th iteration, $y_i$ is the observed number of pinhole events in the *i*th detector pixel, and $c_{ij}$ is the system matrix where a photon emitted from image voxel *j* will be measured in the *i*th detector pixel. The system matrix $c_{ij}$ is calculated by the product of the steric angle from the *i*th detector pixel to voxel *j* through the hole and the probability that a photon interacts with the scintillator pixel.

Furthermore, a PET event is defined as a pair of events deposited at 511 keV by two different HCCs. PET reconstruction was performed by superimposing the lines of response formed by connecting both positions of a PET event. In this study, PET modality was only reconstructed with a simple back projection as the first step and its performance was demonstrated. The quality of PET imaging is expected to further improve by applying reconstruction methods with a filtered back projection or an iterative algorithm. The specific values applied to energy cut during each reconstruction are summarized in Table 4. The number of iterations for the Compton mode and pinhole mode is determined by checking the convergence of the images^48^. The iteration number was 20 for the tri-modal demonstration and 10 for both bottles of drugs and mouse imaging. Note that the Compton, pinhole, and PET events can be selected corresponding to each imaging modality after the measurement.

**Table 4:** Specific values for energy cut applied to each reconfiguration, where $E_f$ and $E_r$ correspond to the energy deposit in front detector and rear detector, respectively.

Targeting source	Targeting energy (keV)	Reconstruction mode	Energy cut (keV)
Cs-137	662	Compton	$20<E_f<80$, $607<E_f+E_r<717$
Am-241	60	Pinhole	$47<E_r<73$
Na-22	511	PET	$471<E_f+E_r<551$
Ga-67	93	Pinhole	$75<E_r<111$
Ga-67	300	Compton	$20<E_f<80$, $273<E_f+E_r<327$
In-111	245	Compton	$20<E_f<80$, $220<E_f+E_r<270$
At-211	79	Pinhole	$69<E_r<89$

### Animal ethics statement

All animal experiments in this study were approved by the animal ethics committees of Osaka University and performed according to the institutional guidelines. We confirm that our work accords with the ARRIVE guidelines.
